# Successful Treatment of Early-Stage Jejunum Adenocarcinoma by Endoscopic Mucosal Resection Using Double-Balloon Endoscopy: A Case Report

**DOI:** 10.1155/2012/521960

**Published:** 2012-07-10

**Authors:** Hirobumi Suzuki, Atsuo Yamada, Hirotsugu Watabe, Yuka Kobayashi, Yoshihiro Hirata, Yutaka Yamaji, Haruhiko Yoshida, Kazuhiko Koike

**Affiliations:** Department of Gastroenterology, The University of Tokyo, 7-3-1 Hongo, Bunkyo-ku, Tokyo 113-8655, Japan

## Abstract

Small bowel adenocarcinoma (SBA) has generally been considered to have a poor prognosis because of nonspecific presentations and difficulties in detection of the disease. The advent of capsule endoscopy (CE) and double-balloon endoscopy (DBE) makes it possible to access to the small intestine for endoscopic interventions. We describe a successful case of early jejunum adenocarcinoma completely resected by endoscopic mucosal resection (EMR) using double-balloon endoscopy (DBE). Early diagnosis and EMR using new technologies such as CE and DBE may improve the recognition of this disease that, at present, has a poor prognosis.

## 1. Background

Small bowel adenocarcinoma (SBA) is a rare malignancy, but has generally been considered to have a poor prognosis, with a five-year survival rate of 48% (stage I-II), 28% (stage III), and 6% (stage IV) [1]. The majority of patients are diagnosed at an advanced stage due to nonspecific presentations and difficulties in detection of the disease. The advent of capsule endoscopy (CE) has allowed examination of the entire small intestine. In addition, double-balloon endoscopy (DBE) makes it possible to confirm the presence of small intestinal diseases and conduct an endoscopic intervention in the small intestine. We report here a successful case of early jejunum adenocarcinoma completely resected by endoscopic mucosal resection (EMR) using DBE.

## 2. Case Report

**Figure 1:**
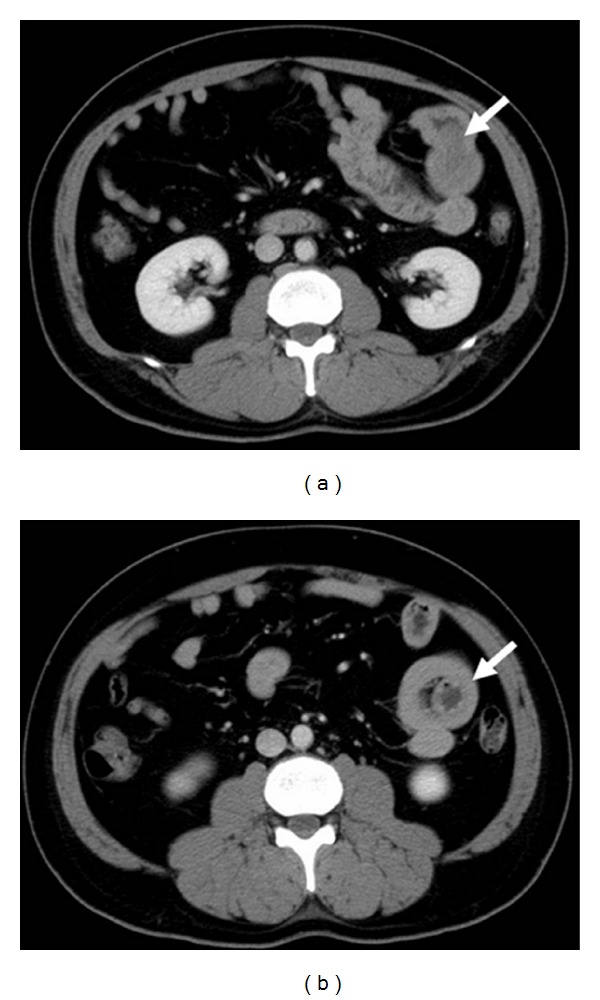
CT findings. (a) Abdominal CT showed the small bowel tumor (arrow). (b) Invagination of a part of the small bowel (arrow).

A 42-year-old man appeared with melena and underwent several examinations, including esophagogastroduodenoscopy (EGD), colonoscopy, and abdominal CT with a contrast agent. Abdominal CT scan revealed a wall thickness in the small intestine (Figures 1(a) and 1(b)). The patient was referred to our hospital.

**Figure 2:**
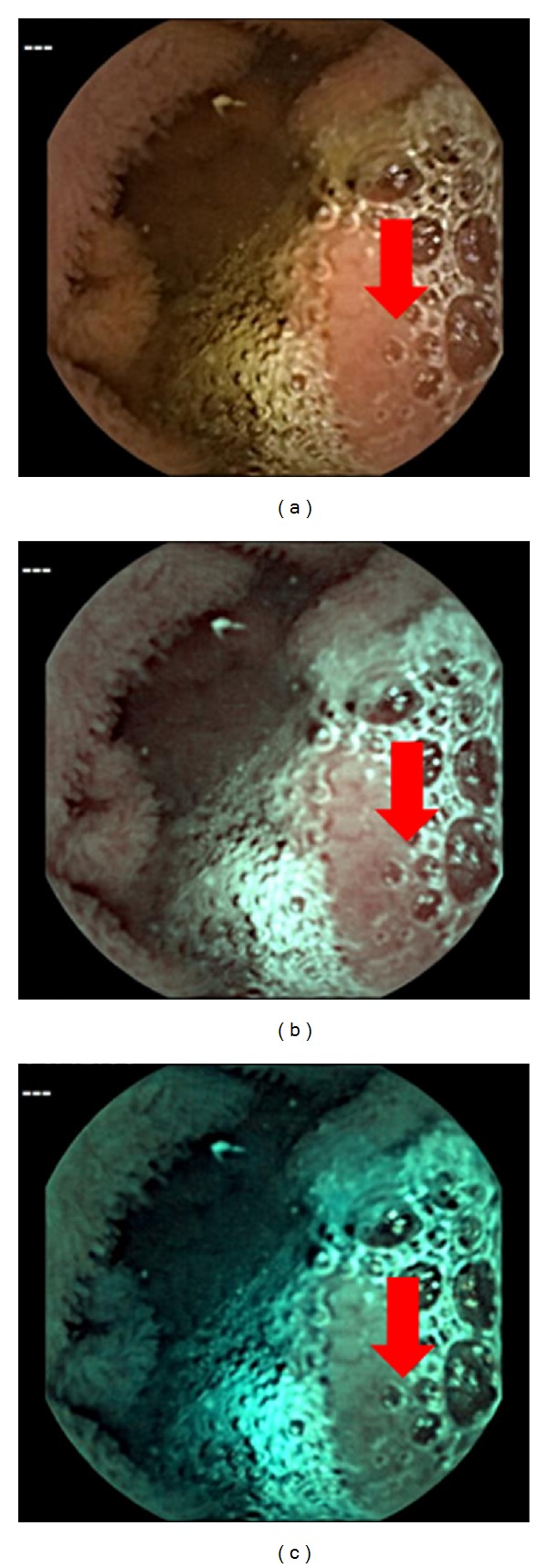
(a) Capsule endoscopy image: CE shows a mass located in the jejunum (arrow). (b) FICE image setting 1 (red 595 nm, green 540 nm, blue 535 nm). (c) FICE image setting 2 (red 420 nm, green 520 nm, blue 530 nm). The mucosal contrast of the tumor was enhanced by FICE settings 1 and 2.

**Figure 3:**
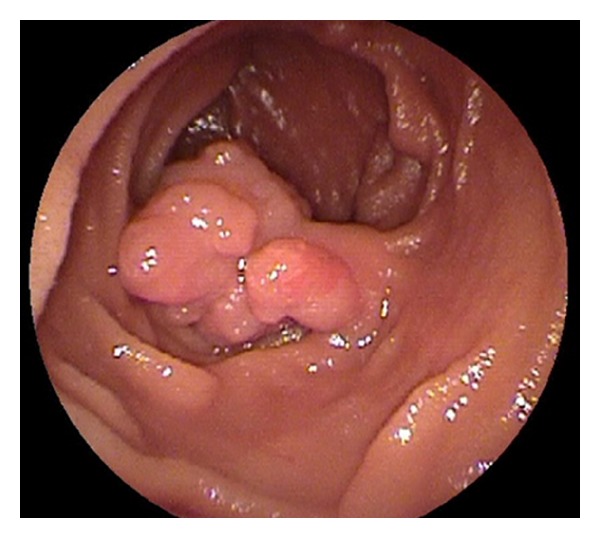
Double-balloon endoscopy showing a 0-Ip polyp in the jejunum. The surface of the polyp was multinodular and villous, and its stalk was slightly reddened.

**Figure 4:**
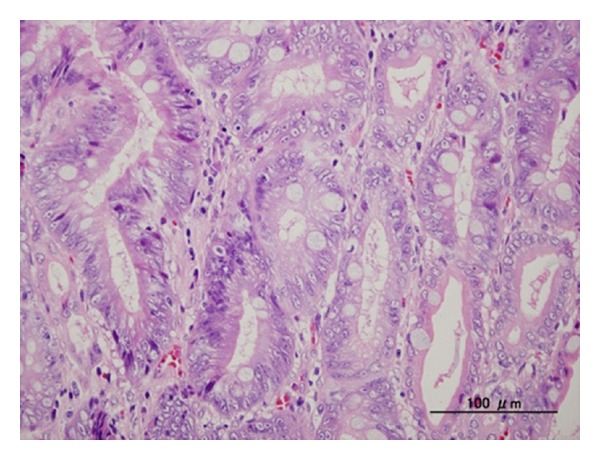
Histological analysis of the EMR specimen showed moderately-differentiated tubular adenocarcinoma with papillary adenocarcinoma. H&E stain. Magnification ×200 (bar = 100 *μ*m).

His hemoglobin level was 11.8 g/dl (normal range 11.3–15.5 g/dl). We conducted a capsule endoscopy and found part of a tumor in the small intestine, suggesting small bowel tumor (Figure 2(a)). In particular, the mucosal contrast of the tumor was enhanced by the effect of the spectral specification of flexible spectral imaging color enhancement (FICE) settings 1 and 2 (Figures 2(b) and 2(c)). In order to investigate the small bowel tumor, antegrade spiral enteroscopy (SE) was conducted [2]. SE showed a multinodular polyp (type Ip) 20 × 15 mm in diameter located about two meters distal to the ligament of Treitz (Figure 3). Biopsy specimen revealed an atypical epithelium. We considered that the lesion was located within the mucosa and decided to perform EMR. We used a DBE (EN-450T5/W, FUJIFILM Medical Co., Ltd., Japan)with an overtube (TS-13140, FUJIFILM Medical Co., Ltd., Japan) and a transparent cap (D-201-11304 Olympus Co., Ltd., Japan) attached to the endoscope tip, which is useful in the colon by pushing draped folds aside or helping luminal orientation at bends by keeping appropriate distance between lens and mucosa [3]. In order to reduce intraluminal gas, a CO_2_  insufflation pump was used during the procedure.It took 12 min to approach the jejunum polyp. Hyaluronic acid at 0.12% (MucoUp, Johnson & Johnson K.K., Japan) was injected into the submucosal layer to lift the surrounding mucosa using a 25-gauge needle (TOP Corp., Japan). A 15 mm electrosurgicaldiameter snare (Snare-Master, Olympus Medical Systems Corp., Japan) was placed around the lifted area. The lesion was strangulated and resected *en  bloc* using blended electric current (Endocut 2 mode ICC200, ERBE, Germany). The electric current output was increased from 60 to 100 W for final resection. Histological examination showed adenocarcinoma (Figure 4). The invasion depth of the carcinoma was limited to the mucosal layer. There was no lymphovascular invasion of carcinoma cells. The lateral and vertical margins of the specimen were negative. The patient was discharged seven days after EMR without any complications, such as perforation and bleeding. Follow-up endoscopy and abdominal CT scan four months after EMR showed no local or remote recurrences.

## 3. Discussion

In the last decade, CE and DBE have enabled visualization of the small bowel [4, 5]. In addition, DBE allows access to the small intestine for endoscopic intervention. In this case, the patient exhibited only nonspecific symptoms. Abdominal CT scan suggested wall thickening, and CE revealed a mucosal change. Spiral endoscopy confirmed the presence of an adenomatous lesion, and, finally, the tumor was completely resected by balloon endoscopy in which we had much more experiences than SE. 

The American Gastroenterological Association (AGA) Institute states that patients with obscure gastrointestinal bleeding (OGIB) need comprehensive evaluation, including CE. In particular, CE is recommended as the third test in the evaluation of GI-bleeding patients after negative bidirectional endoscopy to obtain early diagnosis of small bowel tumors [6]. The frequency of small bowel tumors identified at CE was 2.4–9.6% in patients with a variety of indications, mainly OGIB [7]. 

Imagawa et al. reported that FICE improves the visibility of angioectasia, erosion/ulceration, and various tumors of the small intestine [8]. In particular, tumor visibility was improved with FICE settings 1 and 2. Indeed, these were useful for improving the image quality of the mucosal contrast of tumor.

In contrast, the usefulness of abdominal CT scans in patients with OGIB is unclear. We previously reported that abdominal CT scan is an effective modality to demonstrate duodenal varices in patients with OGIB [9]. This case suggested that abdominal CT scan in patients with OGIB is useful to detect not only duodenal varices, but also early-stage small bowel carcinomas.

To our knowledge, there are few reports of EMR of benign tumors in small intestine such as polyps due to the Peutz-Jeghers syndrome with DBE [10, 11], and none include successful completion of endoscopic resection of pathologically diagnosed SBA. Early diagnosis and EMR using new technologies such as CE and DBE may thus improve the recognition of this disease that, at present, has a poor prognosis.

The present case implies that the combination of several imaging modalities, including CT scan, CE, and spiral or balloon endoscopies, is important to effectively manage a patient with OGIB.
